# Association Between Physical Activity Levels and Mortality and Cardiovascular Disease in Chronic Kidney Disease: A Protocol for a Systematic Review and Meta-Analysis

**DOI:** 10.3390/jcm15051983

**Published:** 2026-03-05

**Authors:** Silvana Patiño-Cardona, Carlos Pascual-Morena, Maribel Lucerón-Lucas-Torres, Marta Carolina Ruiz-Grao, Elena Moreno-Charco, José Alberto Martínez-Hortelano, Irene Martínez-García

**Affiliations:** 1 Health and Social Research Center, University of Castilla-La Mancha, 16071 Cuenca, Spain; silvana.patino@alu.uclm.es (S.P.-C.); mariaisabel.luceron@uclm.es (M.L.-L.-T.); marta.ruiz@uclm.es (M.C.R.-G.); elena.moreno9@alu.uclm.es (E.M.-C.); josealberto.martinez@uclm.es (J.A.M.-H.); 2 Faculty of Nursing, University of Castilla-La Mancha, 02006 Albacete, Spain; irene.mgarcia@uclm.es; 3 Age-ABC Research Group, Health and Social Research Center, University of Castilla-La Mancha, 16002 Cuenca, Spain; 4 Health, Gender, and Social Determinants Research Group, Health and Social Research Center, University of Castilla-La Mancha, 16071 Cuenca, Spain

**Keywords:** exercise, lifestyle, end-stage renal disease, sedentary behavior, prognosis, epidemiology

## Abstract

**Background/Objectives**: Chronic kidney disease (CKD) affects almost 800 million people worldwide. Cardiovascular disease is the main cause of death in this population. Although physical activity is fundamental to systemic health, the evidence regarding its impact on the clinical outcomes of CKD populations is inconclusive. This protocol outlines the methodology for a systematic review and meta-analysis aimed at evaluating the association between physical activity and intensity and all-cause mortality, cardiovascular mortality, and cardiovascular disease. **Methods**: This protocol adheres to PRISMA-P and Cochrane Handbook guidelines and has been registered with PROSPERO (CRD420261302904). A systematic search will be conducted in MEDLINE, Scopus, Web of Science and the Cochrane Library until June 2026. Studies estimating the association between physical activity and all-cause mortality, cardiovascular mortality and cardiovascular disease in populations with CKD will be included. Two independent reviewers will perform study selection, data extraction and quality assessment using the Study Quality Assessment Tool from the United States National Institute of Health tool. The certainty of the evidence will be evaluated using the Grading of Recommendations, Assessment, Development and Evaluation tool. Narrative synthesis and random-effects meta-analysis will be conducted to calculate pooled effect estimates. Random-effects meta-analyses will be performed according to the level of physical activity, and meta-regressions will be used to control for the association with major covariates. Ethical approval is not required for this study. **Results:** The results will provide a comprehensive synthesis of the evidence regarding the use of physical activity as a non-pharmacological intervention to modify CKD progression. **Conclusions:** The findings will be disseminated through peer-reviewed journals and international conferences.

## 1. Introduction

Chronic kidney disease (CKD) is defined as any structural or functional alteration to the kidneys that has persisted for at least three months, with direct implications for the individual’s health [1]. It currently represents a major public health problem, with an estimated global prevalence ranging between 10% and 15%. This places a growing burden on healthcare systems worldwide. According to recent data from the Global Burden of Disease study, CKD affects almost 800 million people worldwide [2]. Its etiology is multifactorial, with diabetes mellitus and hypertension being the most common primary causes, followed by glomerulopathies and genetic disorders [1,3]. The underlying pathophysiological mechanism is characterized by the progressive loss of functioning nephrons, as well as the development of tubulointerstitial fibrosis and glomerulosclerosis. These processes result an irreversible decline in renal function [4].

According to the Kidney Disease Improving Global Outcomes (KDIGO) 2024 guidelines, clinical diagnosis is usually established by confirming an estimated glomerular filtration rate (eGFR) of less than 60 mL/min/1.73 m^2^ or the presence of markers of kidney damage, such as albuminuria [1]. The natural history of the disease tends to involve a progressive and often asymptomatic progression to advanced stages, ultimately resulting in the need for renal replacement therapy [3]. Its main complications include anemia, bone and mineral metabolism disorders, and critically, cardiovascular disease, the main cause of death in these patients [5,6]. Recent interventions, such as the use of sodium-glucose cotransporter type 2 inhibitors (SGLT2i) and non-steroidal mineralocorticoid receptor antagonists (e.g., finerenone), have significantly improved cardiovascular and renal prognosis [7,8]. However, there is considerable variability in the progression of the disease from one individual to another, which is influenced by genetic factors and, crucially, modifiable lifestyle factors [9,10].

In the context of lifestyle, the World Health Organization (WHO) defines physical activity as any bodily movement produced by skeletal muscles that requires energy expenditure. Physical activity is a fundamental pillar for maintaining systemic health and preventing the onset of chronic non-communicable diseases [11]. At a physiological level, physical activity has a positive effect on the body by improving endothelial function, reducing oxidative stress, optimizing glycemic control and decreasing low-grade systemic inflammation, which are all key mechanisms in patients with vascular risk [12]. From a methodological perspective, its quantification in clinical research is inconsistent, which in turn generates discrepancies when classifying patients according to their activity levels. This is due to physical activity measures ranging from subjective standardized questionnaires, such as the International Physical Activity Questionnaire (IPAQ), to objective measurements using accelerometry [13].

However, there is strong consensus from studies of the general population that support a significant reduction in all-cause mortality and the incidence of major cardiovascular events in populations with high levels of physical activity [14]. Based on these benefits, it has been suggested that physical activity can have a positive effect on the progression of CKD. This is thought to be due to its impact on traditional cardiovascular risk factors and its ability to preserve muscle mass and counteract uremia-induced protein catabolism [15].

Despite its biological plausibility, the current literature on patients with CKD yields inconsistent results. Physical activity is known to be associated with a reduced risk of CKD [16]. However, while several observational studies suggest a protective effect, there is a lack of quantified evidence (i.e., systematic reviews and meta-analyses) regarding the relationship between physical activity levels and all-cause mortality, cardiovascular mortality, or cardiovascular disease (CVD) [17]. The main knowledge gap lies in the variability of the measurement instruments used, as well as in the lack of updated syntheses that integrate the most recent evidence. This systematic review and meta-analysis will therefore assess the association between physical activity (both overall and by intensity) and all-cause mortality, cardiovascular mortality and CVD risk in populations with CKD.

## 2. Materials and Methods

### 2.1. Study Design

This systematic review and meta-analysis has been registered with the PROSPERO database (registration number CRD420261302904) [18], and will be conducted in accordance with the PRISMA-P (Preferred Reporting Items for Systematic Reviews and Meta-Analyses Protocols) and Cochrane Handbook guidelines [19,20]. Furthermore, the final report will adhere to the MOOSE (Meta-analyses of Observational Studies in Epidemiology) and PRISMA (Preferred Reporting Items for Systematic Reviews and Meta-Analyses) guidelines [21,22].

### 2.2. Inclusion/Exclusion Criteria

Studies that estimate the association between physical activity and the risk of all-cause mortality, cardiovascular mortality and cardiovascular disease in populations with CKD will be included in the primary studies.

Inclusion criteria will be: (1) Participants: Adults (aged ≥18 years) with a confirmed diagnosis of CKD at any stage. Studies must define CKD according to standard criteria (e.g., KDIGO guidelines) to ensure diagnostic validity. CKD is characterized by an eGFR of less than 60 mL/min/1.73 m^2^ or the presence of kidney damage markers (e.g., albuminuria) that have persisted for at least three months [1]. Studies involving patients on maintenance dialysis (hemodialysis or peritoneal dialysis) or conservative management will be included. (2) Exposure: Physical activity, which is defined as bodily movement that causes energy expenditure, is the main exposure. Intensity will be categorized as light, moderate or vigorous based on metabolic equivalent (MET) thresholds (1.5 to ≥6.0), with sedentary behavior (≤1.5 METs) treated as the lowest physical activity category or reference group if necessary [11]. For studies lacking MET data, the definitions of the original authors will be adopted. Measurements obtained using both objective (e.g., accelerometry and pedometers) and subjective methods (e.g., validated or self-reported questionnaires) will be accepted. (3) Comparator: For categorical comparisons, participants with higher levels of physical activity will be compared to those with lower levels. Sedentary behavior will not be treated as a separate exposure variable, but rather considered and interpreted as the lowest category of physical activity (i.e., the reference group representing inactivity) if necessary, across the included studies. In addition, studies determining the change in risk of outcomes according to increases in physical activity (minutes per week, METs per week, etc.) will be considered. These comparisons will be analyzed separately from categorical comparisons. (4) Outcomes: All-cause mortality, cardiovascular mortality and CVD risk (Major Adverse Cardiovascular Events, or MACE, is a proxy of CVD).

Exclusion criteria will be: (1) Studies involving participants with kidney transplants, acute kidney injury, transient renal dysfunction, or populations where the chronicity of kidney disease (<3 months) cannot be confirmed. (2) Studies evaluating the combined association of physical activity with other lifestyle factors (e.g., diet or smoking) without isolating the risk specifically attributable to physical activity. Studies that do not report risk estimates such as hazard ratio (HR), sub-distribution hazard ratio (SHR), risk ratio (RR) or odds ratio (OR) with their 95% confidence intervals (95% CI) or sufficient data to calculate them will be excluded. No language restrictions will be applied. When there are repeated cohorts in several studies, all studies will be considered in the systematic review, but in the meta-analysis, the study that provides multivariate (adjusted) estimates will be considered first. If both studies provide adjusted estimates, the study with the larger sample size will be considered.

### 2.3. Search Strategy

A systematic electronic search of the MEDLINE (via PubMed), Scopus, Web of Science and Cochrane Library databases will be conducted from their inception to June 2026, and the search will be updated prior to completing the review if necessary. The preliminary search was conducted on 25 January 2026. Grey literature will also be searched for using Google Scholar (web-based search engine), OpenGrey and the Networked Digital Library of Theses and Dissertations. The reference lists of the included studies and any relevant previous reviews will be manually reviewed. The search strategy will be designed using the PECO structure and will combine controlled terms (MeSH and Emtree terms) and free text relating to the following: ‘chronic kidney disease’, ‘physical activity’, ‘sedentary behaviour’, ‘mortality’, and ‘cardiovascular outcomes’. The search will be conducted without language filters, focusing on human studies in adult populations. However, the search results will not be filtered so as not to affect the sensitivity of the search. Finally, Mendeley Desktop (version 1.19.8, Mendeley Ltd., London, UK) was used as the reference manager.

The complete search strategy for each database is detailed in Supplementary Appendix S1.

Two authors will perform the systematic search in duplicate, and any discrepancies will be resolved by consensus or by a third author.

### 2.4. Study Selection and Data Extraction

**Figure 1:**
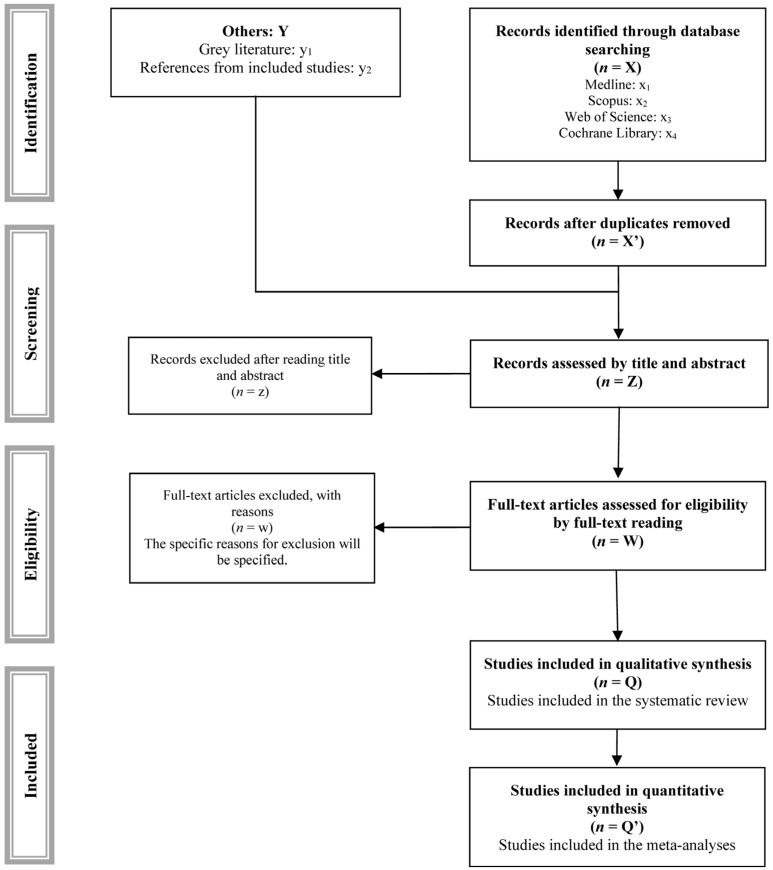
PRISMA flowchart.

After duplicates have been removed, two independent authors will assess the titles and abstracts of the remaining articles to determine their initial eligibility. Any discrepancies between the authors will be resolved by consensus or through the intervention of a third author. In a second phase, the full texts of potentially relevant articles will be examined in accordance with the inclusion and exclusion criteria. The selection process will be documented using a PRISMA flowchart (Figure 1) [22].

Data extraction will be performed by the same two reviewers using a standardized ad hoc data collection form (Table 1 and Table 2). The following information will be extracted: reference (author/year), country, study design, follow-up time, sample size, population characteristics (mean age, percentage of females, baseline eGFR, prevalence of diabetes mellitus/hypertension), covariates used in the adjusted associations, sample size by CKD stages (including percentage of dialysis), method of physical activity measurement, and effect estimates (HR, SHR, RR, OR) for the outcomes of interest.

### 2.5. Study Quality Assessment

The quality of the included studies will be assessed using the Study Quality Assessment Tool from the United States National Institute of Health (National Heart, Lung and Blood Institute) (NHLBI/NIH) tool [23]. This tool evaluates several key methodological domains in order to determine internal validity. The overall quality of each study will be classified as follows: ‘good’ (fewer than two high-risk domains), ‘fair’ (two high-risk domains) or ‘poor’ (more than two high-risk domains). This tool was chosen in preference to other assessment systems (e.g., the Newcastle–Ottawa Scale) because it allows internal validity and risk of bias to be evaluated across different domains, rather than relying solely on a numerical score or quality of reporting. Specifically, it allows reviewers to identify ‘fatal flaws’ that could invalidate the results, ensuring a rigorous assessment of the evidence.

Two authors will independently assess the quality of the evidence, and any disagreements will be resolved by consensus or with the help of a third author.

### 2.6. Grading the Quality of Evidence

The certainty of the accumulated evidence will be independently assessed for each primary outcome using the GRADE (Grading of Recommendations, Assessment, Development and Evaluation) tool [24,25,26]. Following the GRADE framework for observational studies, the certainty of the evidence will initially be classified as ‘low’. This will then be downgraded based on five domains: risk of bias (with particular attention to measurement bias from self-reported physical activity, potential reverse causality and residual confounding factors), inconsistency, indirectness, imprecision and publication bias. Conversely, the certainty of the evidence may be upgraded if a large effect size is observed, if all residual confounding factors are controlled for, or if a dose–response gradient is formally identified through our planned meta-analysis.

### 2.7. Data Synthesis and Statistical Analyses

A narrative synthesis of the findings and study characteristics will be provided. For the quantitative analysis, the effect estimates (i.e., HR, SHR, RR and OR) will be transformed logarithmically. Studies with similar effect estimators (i.e., HR, SHR, RR or OR) and comparison types will be combined using inverse variance analysis. This implies that meta-analyses will be performed of categorical comparisons (high categories compared to the lowest available comparisons, usually in the form of quintiles, quartiles or tertiles), and of linear comparisons (i.e., by increase in physical activity in minutes per day, minutes per week, METs per day, METs per week, etc.). Furthermore, in meta-analyses, different estimators will always be analyzed separately; that is to say, HR, SHR, RR and OR will never be combined in the same analysis.

To ensure comparability across continuous variables, the effect estimates reported per standard deviation (SD) increase will be rescaled to a uniform absolute unit (e.g., per 10 MET-hours/week or an equivalent time metric) by dividing the log-transformed risk estimates and their standard errors by the study-specific SD of the exposure. To explore potential linear and non-linear dose–response relationships between physical activity levels and outcomes, we will apply the method proposed by Greenland and Longnecker, followed by restricted cubic splines [27]. This dose–response meta-analysis will only be conducted if sufficient data are available (i.e., studies reporting at least three quantitative exposure categories, including the reference group, with the corresponding number of cases and total participants or person-years). If the available data do not meet these requirements, the quantitative synthesis will be restricted to predefined categorical comparisons.

Due to the anticipated heterogeneity in the studies’ clinical and methodological approaches, a Hartung–Knapp–Sidik–Jonkman random-effects model will be used for all meta-analyses [28]. This method was selected as the primary estimator because it produces more adequate confidence intervals than the DerSimonian–Laird method, particularly when the number of studies is small. A quantitative meta-analysis will only be conducted if at least three studies provide usable data for a specific outcome. The results will be presented in the form of forest plots with 95% CI. Heterogeneity will be assessed using the *I*^2^ statistic, which is interpreted as follows: ‘not important’ (0–30%), ‘moderate’ (30–50%), ‘substantial’ (50–75%) or ‘considerable’ (75–100%) [20,29], and the between-study variance (τ^2^) will be reported to quantify absolute heterogeneity. Furthermore, 95% prediction intervals will be calculated to estimate the likely range of effect sizes in a future study, providing a more comprehensive interpretation of heterogeneity beyond the *I*^2^ statistic. With regard to competing risk models, we will explicitly extract and prioritize SHR for cardiovascular outcomes, provided these are reported by the primary studies. As previously stated, HR and SHR estimates will be analyzed separately to avoid bias in the pooled effect sizes.

If there are 10 or more studies available, publication bias will be assessed visually using funnel plots and statistically using the Egger test, with a *p* < 0.10 indicating bias [30]. The trim-and-fill method will also be used to estimate the impact of any missing studies [31]. Sensitivity analyses will be performed using the leave-one-out technique to assess the robustness of the overall results. A subgroup analysis will also be performed depending on CKD stage, dialysis status, geographical region, whether objective or subjective measures were used in measuring physical activity, and according to the study design (prospective vs. retrospective). Furthermore, to assess the impact of study quality on the pooled estimates, sensitivity analyses will be conducted. These will involve stratifying the results according to study quality or excluding those classified as ‘poor’. This will enable us to establish whether the observed associations are robust, or whether they are influenced by studies with significant methodological limitations. Additionally, Galbraith plots will be used to identify any atypical studies or outliers that may be contributing excessively to heterogeneity [32]. Meta-regressions will be performed when 10 or more studies are available, taking into account the following covariates: age, percentage of females, eGFR and study duration.

All statistical analyses will be performed using Stata v.18.0 (StataCorp, College Station, TX, USA) software, with a *p* < 0.05 considered to be statistically significant.

### 2.8. Study Status

Preliminary searches have currently been conducted to pilot the study selection strategy, eligibility criteria and data extraction procedures. The final search strategy and data extraction form will be developed based on these findings. The formal database search and study selection were carried out on 25 January 2026, although this search will be updated before the main study is completed in June 2026. Subsequently, the quality assessment and final data analysis will be performed.

### 2.9. Patients and Public Involvement

Patients and the public were not involved in the design or conception of this study.

### 2.10. Ethical Considerations

Approval from an ethics committee is not required to conduct this study because no new human participants will be included. This systematic review and meta-analysis will synthesize the available evidence on the relationship between physical activity levels and all-cause mortality, cardiovascular mortality and CVD risk in people with CKD.

## 3. Discussion

The aim of this systematic review and meta-analysis is to assess the association between physical activity levels and the risk of mortality and cardiovascular events in individuals with CKD. Due to the variety of measurement tools used (e.g., self-report questionnaires versus accelerometry) and the variability in CKD stages, addressing methodological heterogeneity is a main aim of this review. Synthesizing these data will provide the evidence base necessary to assess the potential role of physical activity in the clinical management or progression of CKD.

With regard to all-cause mortality, this study aims to assess the potential association between physical activity and its levels with all-cause mortality. Stratified analysis by activity level (high, moderate or light physical activity compared to no activity) is of critical clinical interest. While high-intensity activity is often emphasized, evaluating the ‘light versus no activity’ comparison is particularly pertinent to real-world clinical practice given the high prevalence of frailty and sarcopenia in this population [33,34,35]. Investigating the relationship between low-intensity physical activity and survival outcomes aims to provide evidence that could inform the setting of achievable therapeutic goals, such as ‘breaking sedentary behaviour’.

With regard to cardiovascular mortality, this review will explore whether the associations of physical activity differ from those observed in all-cause mortality when considering the pathophysiology of inflammation and endothelial dysfunction inherent in uremia [36,37,38]. A level-based analysis will be conducted to assess potential threshold associations or linear dose–response relationships. Clarifying whether moderate physical activity is sufficient to significantly reduce the risk of cardiovascular mortality could help to establish realistic and safe therapeutic goals for this high-risk population, balancing the benefits of exercise against the risk of adverse events.

Similarly, the analysis of CVD risk will focus on non-fatal events such as acute myocardial infarction, stroke and heart failure. The planned stratification by activity level aims to determine whether it is possible to identify a minimum ‘preventive dose’. Should the analysis reveal different levels of incremental benefit across activity categories, these findings could inform the future evaluation of cardiorenal rehabilitation programs.

The planned secondary and sensitivity analyses will enhance our findings. Meta-regressions by age and sex will assess whether potential associations are homogeneous or if specific demographic subgroups might benefit more from the intervention, thus facilitating personalized medicine approaches. Furthermore, advanced statistical techniques, such as the trim-and-fill method and Galbraith plots, will be employed to rigorously evaluate publication bias and sources of heterogeneity. From clinical and research perspectives, this study also aims to highlight the current state of physical activity reporting. If the review identifies a predominance of relative metrics (e.g., tertiles/quartiles) over absolute units (e.g., minutes or MET-hours), this will emphasize the need for future research to adopt continuous, objective metrics. This transition is necessary to enable the precise prescription of physical activity in clinical guidelines, similar to the way in which pharmacological dosing is prescribed.

### Limitations

The inherent limitations of the primary data in this review must be acknowledged. The main limitation is the risk of reverse causality in observational studies, whereby low physical activity may result from health deterioration rather than being its cause. Furthermore, recall bias from self-reported questionnaires can introduce imprecision in exposure levels. Furthermore, residual confounding factors cannot be entirely ruled out, as unmeasured variables such as genetic predisposition or dietary habits may affect both physical activity levels and clinical outcomes. We also acknowledge the risk of survivor bias, which is particularly relevant in populations with CKD, where high mortality rates may result in the inclusion of healthier individuals who are able to maintain higher activity levels. Finally, immortal time bias is a significant challenge in observational studies of physical activity. If the period between the baseline assessment and the subsequent assessment of activity levels is not properly accounted for, the protective effect of physical activity may be overestimated. In addition, methodological heterogeneity resulting from the combination of objective and subjective measures, along with inconsistencies in reporting units (categories versus continuous variables), makes it difficult to define accurate dose–response curves. Finally, the generalizability of the results could be limited by clinical variability according to the stage of CKD and the risk of omitting highly specific literature. Where quantitative data are insufficient, a narrative synthesis will be used. To mitigate these issues, Cochrane Collaboration standards and the PRISMA statement will be strictly applied.

## Figures and Tables

**Table 1:** Ad hoc table for extracting data from the studies included in the systematic review.

Reference	Country	Design	Length	Sample Size	Population Characteristics	Tool of Measurement	Comparison	Outcomes Included
Author and year	Countries where the study was carried out	Prospective, retrospective, case–control	Duration of the study	Total sample size	Comorbidities, CKD stages	Accelerometry or questionnaire	Comparison studied (by percentiles, or linear)	All-cause mortality, cardiovascular mortality, CVD.

Abbreviations: CVD—cardiovascular disease risk.

**Table 2:** Findings of the systematic review.

Reference	Main Comparison	Subgroup Comparison	All-Cause Mortality (95% CI)	Cardiovascular Mortality (95% CI)	Cardiovascular Disease (95% CI)
Author and year	Comparison “Active vs. Inactive”, or by increase in time of physical activity, or by increase in scale of physical activity.	Categorization of the level of physical activity as reported by the authors or, failing that, assignment of the level of physical activity according to the categories used by the authors.	Association in HR, SHR, RR or OR.	Association in HR, SHR, RR or OR.	Association in HR, SHR, RR or OR.

Abbreviations: 95% CI—95% confidence interval; HR—hazard ratio; SHR—sub-distribution hazard ratio; OR—odds ratio; RR—risk ratio.

## Data Availability

Data are available upon reasonable request to the corresponding author.

## Supplementary Materials

The following supporting information can be downloaded at: https://www.mdpi.com/article/10.3390/jcm15051983/s1, Appendix S1: Search Strategy.

## Abbreviations

The following abbreviations are used in this manuscript:

95% CI	95% confidence interval
CKD	Chronic kidney disease
CVD	Cardiovascular disease
eGFR	Estimated glomerular filtration rate
GRADE	Grading of recommendations, assessment, development and evaluation
HR	Hazard ratio
IPAQ	International physical activity questionnaire
KDIGO	Kidney disease improving global outcomes
MACE	Major adverse cardiovascular events
MOOSE	Meta-analyses of observational studies in epidemiology
OR	Odds ratio
PRISMA	Preferred reporting items for systematic reviews and meta-analyses
PRISMA-P	Preferred reporting items for systematic reviews and meta-analyses protocols
RR	Risk ratio
SGLT2i	Sodium-glucose cotransporter type 2 inhibitors
SHR	Sub-distribution hazard ratio
WHO	World Health Organization
