# Supplementary material for: Association Between Physical Activity Levels and Mortality and Cardiovascular Disease in Chronic Kidney Disease: A Protocol for a Systematic Review and Meta-Analysis
Source: J Clin Med. 2026 Mar 5;15(5):1983. doi: 10.3390/jcm15051983 (PMC12985847; doi:10.3390/jcm15051983)
Supplement: Supplementary file 1 [file jcm-15-01983-s001.zip › jcm-4177697-supplementary.pdf]

Appendix S1. Search Strategy.

- Medline, Scopus, Web of Science, and Cochrane Library

("kidney disease" OR "chronic kidney disease" OR "chronic renal disease" OR "chronic kidney insufficiency" OR nephropathy OR "renal impairment") AND ("physical activity" OR "sedentary behaviour" OR “sedentary behavior”) OR ("risk" OR "mortality" OR “cardiovascular mortality” OR "survival" OR cardiovascular OR “cardiovascular disease” OR “major adverse cardiovascular event”)

- Grey literature

Not specified (open search).
